# Aspirin—Another Type of Headache it Prevents

**DOI:** 10.1161/JAHA.113.000111

**Published:** 2013-04-24

**Authors:** R. Loch Macdonald

**Affiliations:** 1 Division of Neurosurgery, St. Michael's Hospital, Labatt Family Centre of Excellence in Brain Injury and Trauma Research, Keenan Research Centre of the Li Ka Shing Knowledge Institute of St. Michael's Hospital, Department of Surgery, University of Toronto, Ontario, Canada (L.M.)

**Keywords:** Editorial, cerebral aneurysm, aspirin, subarachnoid hemorrhage, ferumoxytol

## Introduction

Aspirin is a trade name, owned by Bayer, for acetylsalicylic acid (ASA). Although this name is considered generic in many countries, such as the United States, it is not in Canada. It was synthesized as early as 1853 by the French chemist, Charles Gerhardt, but the first synthesis using salicin derived from willow leaves is usually attributed to Felix Hoffmann at Bayer in Germany in 1897.^1–2^ There is controversy, however, as to whether Arthur Eichengrun actually did the work at Bayer. Its basic mechanism of action was discovered by Vane, Bergstrom, and Samuelsson in the 1970s, which resulted in their obtaining a Nobel prize in 1982. ASA, like most drugs, has several actions, but its most well characterized is that it irreversibly acetylates cyclooxygenase‐1 (COX‐1) and COX‐2, also known as prostaglandin H synthases 1 and 2.^3^ These enzymes convert arachidonic acid to prostaglandin H, which is the first step in biochemical synthesis of the prostanoids. COX‐1 is constitutively expressed whereas COX‐2 is induced in, for example, macrophages and tissues during inflammation. Inhibition of COX‐1, which is found predominately in platelets and which produces prothrombotic, vasoconstricting thromboxane A_2_, is achieved with relatively low doses given once a day. For inhibition of COX‐2, which is involved in pain and inflammation, higher doses need to be given more frequently. Vascular endothelium produces prostaglandin I_2_ via COX‐2, which is vasodilatory and antithrombotic. The beneficial effects of low doses of ASA against cardiovascular disease are probably due to this selective inhibition of COX‐1 in platelets. Although the plasma half‐life of ASA is only 20 minutes, its action on platelets lasts for days. The dose to inhibit COX‐1 ranges from about 30 mg/day up to about 100 mg/day. Higher doses cause more gastrointestinal side effects and can cause more bleeding complications. Other actions of ASA may include inhibition of other platelet functions, increasing fibrinolysis and inhibition of coagulation.^3^ The anti‐inflammatory effects, which are the subject of this review, are probably related to another set of actions such as the formation of nitric oxide radicals and the modulation of inflammatory signaling pathways. These effects also may be mediated by the main ASA metabolite, salicylic acid.

Hasan and colleagues^4^ describe a novel effect of ASA in a human study reported recently in the *Journal of the American Heart Association*. They imaged 11 patients with unruptured intracranial aneurysms using ferumoxytol‐enhanced magnetic resonance imaging (MRI) and then randomly allocated them to treatment with 81 mg/day of ASA or to no treatment at all. When the aneurysms were surgically repaired 3 months later, the walls of the aneurysms from patients treated with ASA showed lower expression of COX‐2, microsomal prostaglandin E_2_ synthase 1, and macrophages. This correlated with reduced ferumoxytol enhancement on MRI before surgery in treated patients. The mechanism by which ASA would reduce COX‐2 and macrophages is unknown but there is some evidence that the association of ASA use with reduced colorectal cancer is related to its antiplatelet effects.^5^ As Hasan et al speculate, inhibiting platelet activation could reduce endothelial injury and the subsequent aneurysm wall injury and inflammation.

The basis for this work was the authors' prior post hoc analysis of data from the International Study of Unruptured Intracranial Aneurysms, which found that patients taking ASA had a lower risk of aneurysm rupture.^6^ The walls of ruptured human aneurysms have been compared to unruptured aneurysms and found to have higher immunohistochemical staining for COX‐2 and microsomal prostaglandin E_2_ synthase 1.^7^ Experimental studies also reported that macrophages seem to contribute to development of aneurysms in mice because depletion of macrophages reduces aneurysm formation.^8^ Hasan et al succinctly review a theoretical pathway by which low wall shear stress in the aneurysm leads to monocyte infiltration, inflammation, and potential aneurysm rupture.

There are several interesting and novel aspects of this study. The use of ASA is perhaps somewhat counterintuitive to neurosurgeons, neurointerventionists, and others who treat patients with unruptured aneurysms. ASA would be expected to worsen the outcome should the aneurysm rupture since there ought to be more bleeding from the aneurysm. Juvela^9^ reported that patients who were taking ASA when they had subarachnoid hemorrhage (SAH) had a lower risk of delayed cerebral infarction, although overall outcome was not reported. This question will need to be addressed in future studies. The authors are to be commended for their use of cell‐targeted imaging. Another question is the extent to which the study was blinded, given the lack of a placebo control. Also, neurosurgeons might be somewhat nervous to operate on patients only 1 day after stopping ASA as was done in this study. Finally, the measurements of ferumoxytol enhancement and immunohistochemistry are qualitative at this point.

What is the clinical significance of these findings? The study uses a surrogate marker of inflammation, which is uptake of ferumoxytol by macrophages. How this relates specifically to inflammation and whether the type of inflammation imaged here related to macrophages is good or bad for aneurysms is not exactly known. Macrophages are suggested to be mediating a deleterious process in these aneurysms but this is not proven. Finally, the design of a study to address whether ASA prevents SAH from aneurysms, is going to need to be addressed as important work like this study becomes available to support the use of drugs to prevent aneurysm rupture.
